# Fatal Attraction: How Bacterial Adhesins Affect Host Signaling and What We Can Learn from Them

**DOI:** 10.3390/ijms16022626

**Published:** 2015-01-23

**Authors:** Daniel H. Stones, Anne-Marie Krachler

**Affiliations:** Institute of Microbiology and Infection, School of Biosciences, University of Birmingham, Birmingham B15 2TT, UK; E-Mail: d.stones@bham.ac.uk

**Keywords:** adhesion, adhesin, cell-signaling, host–pathogen interaction, bacterial attachment, anti-adhesion therapy

## Abstract

The ability of bacterial species to colonize and infect host organisms is critically dependent upon their capacity to adhere to cellular surfaces of the host. Adherence to cell surfaces is known to be essential for the activation and delivery of certain virulence factors, but can also directly affect host cell signaling to aid bacterial spread and survival. In this review we will discuss the recent advances in the field of bacterial adhesion, how we are beginning to unravel the effects adhesins have on host cell signaling, and how these changes aid the bacteria in terms of their survival and evasion of immune responses. Finally, we will highlight how the exploitation of bacterial adhesins may provide new therapeutic avenues for the treatment of a wide range of bacterial infections.

## 1. Introduction

Bacteria are continually evolving mechanisms in order to successfully colonize and survive in many different environmental conditions. For some bacteria these adaptations have enabled them to thrive within the human body. Both pathogenic and commensal bacteria display a wide range of surface bound and secreted molecules that are able to aid their colonization of the host. Arguably, one of the most important characteristics of bacterial colonization is adhesion. Adhesion not only allows bacteria to colonize through simply sticking to host cell surfaces and thus generating a stable platform on which to grow, but is also required for the release of toxins and virulence factors that drive infection. How different bacterial populations use the multiple adhesins present on their surface (Table 1) and how they bind to specific cell receptors located in niche environments within the host can also influence the type of disease caused by a particular organism. The formation of biofilms, which are known to increase antibiotic resistance and reduce clearance from the host, is also highly dependent upon bacterial adhesion molecules. Further to this, adhesion of bacteria to host cell surfaces can affect not only bacterial cell signaling but also lead directly to changes in host cell signaling, enabling bacterial spread and evasion of host immune responses.

It is therefore clear that adhesion remains an integral feature throughout the course of bacterial infections. While the topic of bacterial adhesion and to some extent the effect this has on host cell signaling has been reviewed previously [1,2], in this review we aim to summarize the key points related to the different mechanisms of bacterial adhesion and highlight recent advances in the field, with an emphasis on the effects adhesion can have on host cell signaling and finally how these interactions may be exploited in terms of novel therapies for a broad range of bacterial infections, while avoiding off-target effects on the host.

## 2. Bacterial Adhesin Classes and Their Ligands

### 2.1. Integrin and Fibronectin Binding Proteins

Integrins represent a highly conserved group of heterodimeric transmembrane glycoproteins that are essential for many cell–cell and cell–matrix interactions. The collagen binding integrins in particular have been shown to be conserved throughout the metazoan tree of life and form an essential component of multi-cellularity in animals [3,4,5]. Due to this wide spread presence throughout the animal kingdom and the fact that integrin signaling facilitates many essential cell signaling cascades, including those involved in cell adhesion and cytoskeletal organization, many bacterial species have evolved adhesion mechanisms that interact either directly or indirectly with host integrin receptors.

**Figure 1:**
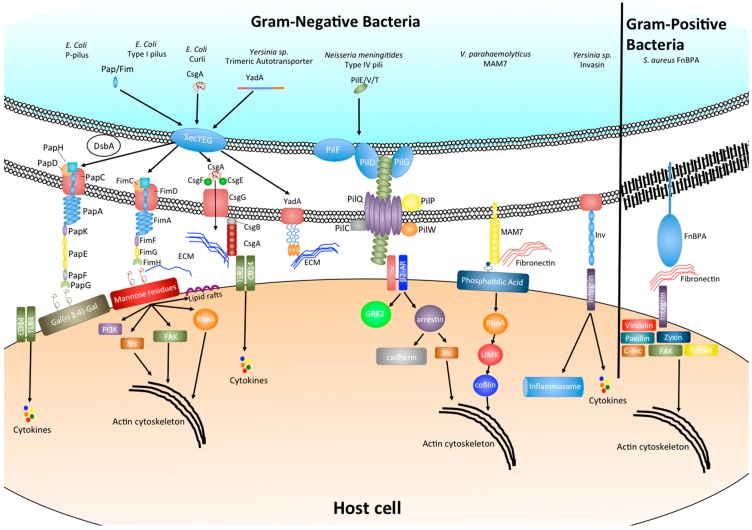
Bacterial adhesins and their effect on host cell signaling.

Fibronectin binding proteins (FnBPs) make up a diverse group of surface adhesins that bind to the extracellular matrix (ECM) protein fibronectin. As such, they are a subclass of a large family of bacterial adhesins referred to as microbial surface components recognizing adhesive matrix molecules, or short, MSCRAMMS [6]. In the case of the Gram-positive bacterium *Staphylococcus aureus* this interaction with fibronectin within the ECM is able to facilitate bacterial binding to the host cell surface by exploiting fibronectins binding to the host cell integrin α_5_β_1_ (Figure 1). The binding of *S. aureus* FnBPA to integrin α_5_β_1_ via fibronectin bridging has been shown to facilitate bacterial uptake into host cells [7]. In addition the *Streptococcal* FnBP Sfbl/F1 has also been shown to mediate invasion of epithelial cells [8,9]. Although the binding of FnBPs to fibronectin has been reported to be a strong interaction (~2.5 nN), possibly due to the fact that a single FnBP can bind up to 9 fibronectin molecules [10,11], the importance of FnBPs during infection when comparing either wild type or FnBP mutant strains *in vivo* has been variable. It has been suggested that this may be due to the typically wide range of diseases caused by these organisms and the prevalence of additional virulence factors in some circumstances may have redundant roles [12]. However a more recent study has demonstrated that FnBPs are essential for biofilm formation in *S. aureus* strain LAC, a methicillin resistant clinical isolate [13].

**Table 1:** Bacterial adhesins and their ligands.

Organism	Adhesin	Ligand	Function	Refs.
*S. aureus*	Clumping factor A (ClfA)	Fibrinogen γ-chain	Adhesion and immune evasion	[14,15]
	Clumping factor B (ClfB)	Fibrinogen α-chain, keratin 10 and loricrin	Adhesion to desquamated epithelial cells	[15]
	FnBPA/FnBPB	Fibronectin, Fibrinogen γ-chain and elastin	Adhesion to ECM, biofilm formation	[15]
	Collagen adhesin (Cna)	Collagen, complement C1q	Adhesion, complement evasion	[15]
*Streptococcal* sp.	Sfbl	Fibronectin	Adhesion	[8,9]
*Yersinia* sp.	Invasin	β1-integrin	Adhesion, internalization	[16]
	Trimeric autotransporter YadA	Fibronectin, Collagen	Adhesion, internalization	[17]
	Ail	Fibronectin, Laminin, C4bp, complement H	Yop delivery, adhesion, internalization, serum resistance	[18,19]
*E. coli*	CU P-pilus	Gal(α1-4)gal containing receptors	Adhesion, immune response	[20]
	CU type I pili	Mannose containing glycoproteins	Adhesion, inflammation	[21,22]
	Afa/Dr	Collagen, hDAF, CEACAMs	Adhesion, inflammation	[23]
	Curli	Fibronectin, laminin	Biofilm formation, invasion, inflammation	[24]
	Trimeric autotransporter Antigen 43	Unknown	Aggregation	[25]
*N. meningitidis*	Type IV pilus	Unknown	Adhesion, aggregation, motility, DNA transfer	[26]
*M. tuberculosis*	Mtp amyloid	Laminin	Adhesion, colonization	[27]
	MCE1a	Unknown	Adhesion, invasion	[28,29]
*V. parahaemolyticus*	MAM7	Phosphatidic acid, fibronectin	Adhesion, invasion	[30]
*H. pylori*	Type IV pilus	β5-Integrin	Gastrin production, increases acidity	[31]
	BabA	Lewis B antigen	Adhesion, inflammation	[32]
*L. rhamnosus* GG	SpaCBA pilus	Mucus	Adhesion, immunomdulation	[33,34]
*Salmonella* sp.	FliC	Cholesterol	Adhesion, biofilm formation	[35]
	PefA	Lewis X blood group antigen	Adhesion	[36]
	Type I pilus FimH	Mannose containing glycoproteins	Adhesion	[37]

FnBP: fibronectin binding proteins; ECM: extracellular matrix; CU: Chaperone-usher.

Bacteria can also adhere to and internalize into host cells by direct interaction with integrins. The *Yersinia* protein invasin facilitates initial adhesion of the bacterium and binds with high affinity to β1-integrin receptors found on the surface of M cells [16]. However, following initial attachment and invasion, the expression of invasin is reduced and adhesion is maintained by the adhesins YadA and Ail which mediate serum resistance and promote tight adherence to ECM proteins fibronectin and collagen (Figure 1) [17,19]. The mechanism of invasin-induced internalization will be discussed below.

### 2.2. Chaperone-Usher Pili: P Pili and Type I Pili

Chaperone-usher (CU) pili are some of the most well-characterized bacterial adhesins. They form long proteinaceous strands made up of several subunits, which extend from the surface of many Gram-negative as well as some Gram-positive bacteria and can be divided into a “tip” and a helically wound “rod” like domain [20,21]. Due to the fact that certain pili can also be utilized for the transfer of DNA during conjugation, those that are used exclusively for adhesion to host cell surfaces are often referred to as fimbriae. The first fimbria to be described was the P-pilus, which is expressed under the control of the *pap* operon by uropathogenic *E. coli* (UPEC) and interacts with the α-d-galactopyranosyl-(1-4)-β-d-galactopyranoside moiety of glycolipids present on upper urinary tract cells via the tip adhesion subunit PapG (Figure 1). Variations in PapG can also recognize different but related Gal(α1-4)gal receptors differentially distributed within the host as well as within populations and is thought to drive tissue and host specificity [38]. The biogenesis of the P-pilus has been widely studied in molecular detail and is the archetype of chaperone-usher pilus formation. Individual unfolded subunits are transported into the periplasm by the general secretory pathway [39] where they first undergo disulphide bond formation by DsbA. The subunits are then further stabilized and transported by the chaperone PapD to the outer membrane usher PapC which forms and extends the pilus, starting at the tip, via donor strand exchange [21].

Type I pili represent another class of heteropolymeric fimbriae present on the surface of pathogenic *E. coli* (UPEC and DAEC) and are encoded by the *fim* operon. Similar to the P-pilus, the type I pili are formed through a CU pathway comprising FimC as the periplasmic chaperone and FimD as the outer membrane usher (Figure 1) [40]. The adhesin tip of the fimbria is formed by the FimH subunit which binds mono- and tri-mannose containing glycoproteins. Structural and biophysical analysis of the type I and P-pili have demonstrated that binding of the tip adhesins to their respective ligands is via a catch bond (a bond whose strength is increased by a force such as shear stress) and that the regulation of binding strength can be controlled by uncoiling of the helically wound rod domain [41]. In addition, recent evidence has also implicated FimH as a key factor in influencing virulence. It has been demonstrated that through alteration of adhesin conformation by point mutations in FimH of Crohn’s disease associated adherent-invasive *E. coli* results in enhanced intestinal inflammation by an unknown mechanism [22].

### 2.3. Type IV Pili

The type IV pili are another group of polymeric surface organelles that are among the most wide spread throughout Gram-positive bacteria, Gram-negative bacteria as well as Archaea and have been previously reviewed in depth elsewhere [26]. Unlike CU pili, the precise biogenesis and adhesion properties of type IV pili are still poorly understood, partly due to the large number of different proteins involved in pilus formation [42] and also the high functional diversity exhibited by many type IV pili including adhesion, aggregation, DNA transfer, electron transfer and motility. Despite this, studies have so far determined that type IV pilus formation involves the translocation of pre-pilins across the inner membrane where pre-pilin peptidase recognizes and cleaves a conserved *N*-terminal type III signal sequence, thus forming a mature pilin subunit. Upon release from the inner membrane, the pilin subunit is then assembled into a fiber via an ATPase dependent manner along with several accessory protein molecules (Figure 1), [43]. In *Neisseria meningitidis* the ATPase PilF catalyzes the extension of the pilin fiber and PilT is involved in the retraction of the pilus through the bacterial cell wall while the pilus remains bound to the target surface [44]. This interplay between elongation and retraction has been shown to depend on levels of PilT and force mediated elongation, which can lead to altered interaction between the bacteria and host cells by increasing pilus tension [45]. More recent studies have also highlighted that the number of pili on the surface of *N. meningitidis* also can alter the interaction and cell signaling of host cells [46,47].

### 2.4. Adhesive Amyloids

Amyloids are insoluble polymeric protein fibril-like structures that share a common cross stacking of folded β-sheets. They were first recognized in human diseases such as Alzheimer’s, Huntington’s and prion encephalopathies but have since been found to be extremely wide spread in nature and display a broad range of functional diversity [24]. Curli are probably the best described class of functional amyloids and are produced by enteric bacteria such as *E. coli*, *Salmonella*, *Citrobacter*, and *Shewanella*. Amyloid fibers have also been found in 5%–40% of species isolated from natural biofilms [48]. In *E. coli* two distinct operons are involved in curli formation, the *csgBAC* operon and the *csgDEFG* operon. The *csgDEFG* operon encodes the soluble transcription regulation subunit CsgD as well as chaperones CsgE and CsgF which co-ordinate with CsgG to form a distinct secretion system. The secretion system then transports curli subunits CsgA and CsgB to the cell surface where CsgB nucleates CsgA into the highly stable fibril polymer (Figure 1). Recent structural evidence has highlighted that CsgG forms an un-gated, non-selective protein secretion channel that along with CsgE restricts the conformational space within the channel by forming an encaging complex. This caging generates an entropic free-energy gradient over the channel and allows for protein translocation across the membrane through an entropy driven, diffusion-based method [49]. The main role of amyloid fiber adhesion for most bacterial species is during biofilm formation in which they help to increase biofilm stability through interactions with host ECM proteins such as fibronectin and laminin and also enhance resistance to protease degradation. Mtp amyloid fibers from *Mycobacterium tuberculosis* have been shown to bind to laminin in the ECM and contribute to bacterial adhesion and colonization [27].

### 2.5. Autotransporters

The autotransporters are a diverse family of outer membrane and secreted proteins that are found in many Gram-negative bacteria and form a monomeric or trimeric structure. In most cases they facilitate adhesion to host cell surfaces and ECM as well as bacterial aggregation and biofilm formation (Figure 1). All autotransporters share conserved structural features, including an *N*-terminal signal sequence which enables secretion of the protein across the inner membrane via the general secretory pathway, a conserved *C*-terminal translocation domain which inserts into the outer membrane, and a variable passenger domain that can either be free or anchored to the cell surface and influences the adhesive properties of the protein [50,51]. The first trimeric autotransporter to be described was YadA of *Yersinia* sp. [52]. YadA from different *Yersinia* sp is thought to adhere to different ECM components [17]. Despite their wide spread and central role in bacterial pathogenesis the precise molecular mechanisms of action for many of the autotransporter proteins are still poorly defined. Recent evidence from the structure of Antigen 43, an autotransporter from uropathogenic *E. coli*, has highlighted a twisted l-shape β-helical structure that is proposed to form a molecular “Velcro-like” mechanism of self-association facilitating bacterial clumping [25]. A study evaluating the binding interactions of *Burkholderia cenocepacia* trimeric autotransporters has revealed that homophilic and heterophilic interactions formed by autotransporter BCAM0224 are of a low affinity. This weak adhesion may have biological significance as during colonization of the lung a lower affinity would allow for dynamic interplay between adhesion and movement of the bacteria, thus allowing the pathogen to spread and bind to new sites [53].

### 2.6. Multivalent Adhesion Molecules

The multivalent adhesion molecules (MAMs) are a relatively recent class of bacterial adhesins to be described and participate in high affinity binding during the early stages of infection of a wide range of Gram-negative bacteria [30]. MAMs consist of an *N*-terminal hydrophobic region, followed by either six (MAM6) or seven (MAM7) mammalian cell entry (MCE) domains (Figure 1). While MAM6 and MAM7 molecules are found exclusively in Gram-negative bacteria, single MCE domain containing proteins are more widely conserved and in addition to Gram-negative bacteria, are also found in *Mycobacteria* and some Gram-positive bacteria as well as algae and higher-plants. The MCE domain was first described in *Mycobacteria* where there are four separate operons encoding MCE proteins [29,54]. The vast majority of these are thought to play a role in lipid metabolism [55,56] but Mce1A has been shown to facilitate *M. tuberculosis* adhesion and internalization into non-phagocytic host cells [28,29]. Differences in Mce1A between *M. tuberculosis* and *M. leprae* have been suggested to be a potential mechanism of tissue specific infection of the two species [57]. As mentioned above, in Gram-negative bacteria the number of MCE domains within MAMs is highly conserved to six or seven MCE domains and it has previously been shown that six domains is the minimum number required for efficient binding to host cells [58]. Interestingly, recombinant MAMs with three to five MCE domains in tandem have been found to misfold or result in highly unstable proteins, which reasons why this domain configuration is not seen in nature. However the molecular basis for this observation is still poorly understood. Secondary structure prediction reveals that MAMs are rich in β-strands connected by flexible loop regions; similar to FnBPs. Characterization of *Vibrio parahaemolyticus* MAM7 binding interactions has revealed that the host ligands for MAM7 adhesion are fibronectin and phosphatidic acid (PA) [30]. While many bacterial receptors have been found to bind fibronectin this is the first bacterial adhesin shown to bind directly to lipid ligands within the host cell membrane. The binding to fibronectin was found to be a moderate affinity with an equilibrium dissociation constant (*K_D_*) of 15 μM, however PA binding was found to be much greater with a *K_D_* of 200 nM. A more recent study of this interaction has demonstrated that PA is essential for adhesion to host cells and is mediated mainly by key basic residues in MCE-1, 2, 3 and 4, whereas fibronectin is dispensable and merely acts to increase the rate of host cell binding [58]. The interaction with fibronectin was found to require at least 5 MCE domains and that only a 30 KDa *N*-terminal fragment of fibronectin was needed to facilitate binding. Unfortunately the molecular mechanism of how MAM proteins form protein–protein and protein–lipid interactions simultaneously and the key residues involved are still unknown.

## 3. Effect of Bacterial Adhesion on Host Cell Signaling

The ability to attach to host cell surfaces is evidently a key first step in colonization as this can reduce the ability of clearance from the host through shear stress, however, attachment alone is not enough to establish and maintain an infection. Bacteria have evolved mechanisms of manipulating the surrounding host environment and immune response to aid their spread and survival through alteration of host cell signaling. Whilst this ability in the later stages of infection can be attributed to a myriad of secreted effectors, depending on the bacteria and niche environment, there is accumulating evidence that at the initial stages of the infection many species are able to manipulate host cell signaling directly through the process of adhesion.

As mentioned previously, the integrin family of host cell surface receptors are a key target for adhesion of many bacterial species and normally regulate cell–cell and cell–ECM contacts through a wide range of intra-cellular signaling pathways. This central role of integrins in host cell structure and tissue integrity can be altered in different ways by a variety of bacteria, depending on the type of bacteria and the infection caused. The binding of *S. aureus* FnBPs to host cell β1 integrins via a fibronectin linkage leads to integrin clustering and recruitment of focal adhesion like protein complexes which include cell signaling molecules such as vinculin, paxillin, zyxin, tensin, FAK and c-Src. This results in downstream signaling and a re-organization of the actin cytoskeleton, facilitating invasion of host cells [3]. As well as effects upon the cytoskeleton, β1-integrin binding by *Yersinia enterocolitica* invasin protein has been shown to be an early trigger for inflammasome activation and interleukin-18 (IL-18) production in intestinal epithelial cells (the main target cell for this pathogen), which suggests that in these circumstances β1-integrin may have evolved a second function as a pathogen recognition receptor. This initial invasin-triggered inflammation is later counteracted by the type III secretion system effector proteins YopE and YopH [59]. The Type IV pilus adhesin, CagL, of *Helicobacter pylori* has recently been shown to induce gastrin production in gastric epithelial cells by adhesion to β5-integrin/integrin linked kinase complexes and downstream signaling through the epidermal growth factor receptor (EGFR), Rapidly Accelerated Fibrosarcoma (Raf) kinase, mitogen activated protein kinase kinase (MEK), extracellular signal regulated kinase (ERK) pathway, thus increasing the acidity of the stomach which can lead to gastric ulcer formation and gastric adenocarcinoma [31]. A second adhesin of *H. pylori*, the blood group antigen binding adhesin BabA, which binds human Lewis (b) surface epitopes, has been shown to induce IL-8 production through adhesion mediated activation of the type IV secretion system [32]. A separate study also found BabA adhesion to cause DNA double strand breaks through an unknown mechanism, again highlighting this pathogen as a strong inducer of gastric inflammation and carcinogen [32,60].

Although integrin binding is a common target for many pathogens to alter actin cytoskeletal organization, recent studies have highlighted alternative cell surface molecules that may also result in downstream effects on the cytoskeleton. Phosphatidic acids make up between 1% and 4% of a cell’s phospholipid content and are key precursors for other phospholipids, regulate membrane curvature and can affect a broad range of signaling molecules [61,62,63,64,65]. Clustering of the adhesin MAM7 of *V. parahaemolyticus* at the host cell surface upon binding to phosphatidic acid has recently been shown to mediate activation of the small GTPase RhoA. The activation in RhoA leads to actin rearrangements, resulting in the redistribution of tight junction proteins and disruption of epithelial integrity. This destruction to the epithelial barrier allows *V. parahaemolyticus* to translocate across polarized epithelial layers [66].

Bacterial adhesins can also elicit immune responses in host tissue, such as the CsgA curlin subunit of Enterobacteriaceae which binds to and activates Toll-like receptor 2 signaling in host cells leading to increased inflammation [67].

## 4. The Potential of Adhesion Inhibition as Novel Infection Intervention

The widespread rise of antibiotic resistance in many clinically significant pathogens is a serious threat to global health and new methods to combat infections need to be developed urgently. Ideally, new therapies will target virulence factors associated with bacterial colonization rather than immediate survival, thus allowing infection attenuation and natural clearance. This targeting method may apply less selective pressure upon the bacterium and would conceivably result in a reduction of the amount of antibiotic resistant strains emerging. As this review has highlighted, adhesion plays an early and integral part in bacterial colonization and survival within the host and as such has been a target for many anti-infection studies, especially in the background of antibiotic resistant strains. The idea of anti-adhesion therapy is not new and has been reviewed previously [68,69] with the first deliberate attempt to block FimH adhesion to mannose containing host cell receptors by using mannoside derivatives [70]. However, despite their obvious appeal, anti-adhesion therapies are still not in mainstream use for the treatment of bacterial infections. One reason for this is that bacteria possess multiple adhesion molecules that are expressed in a time- and tissue-specific manner during the course of an infection and this redundancy presents a real challenge for anyone developing an anti-adhesion therapy. A possible way to counteract this would be to use a cocktail of inhibitors that target multiple adhesion molecules and/or use these inhibitors alongside traditional antibiotic therapy. Another challenge in the field of anti-adhesion therapy is the design of high affinity inhibitors that are able to effectively out compete and remove adherent bacteria from the cell surface, while avoiding interference with endogenous host signaling pathways. This will require a deeper understanding of features within bacterial adhesins required for surface attachment and activation of signaling pathways, which will further work on uncoupling these two functions and design inhibitors which specifically outcompete bacterial pathogens, while avoiding off-target effects. Further structural insight into specific adhesin-host interactions along with the design of multivalent display systems will undoubtedly be needed for the development of new anti-adhesion therapies. However, recent studies are beginning to demonstrate the feasibility of anti-adhesion therapy. Uropathogenic *E. coli* O25b:H4-ST131, a multi-drug resistant strain which causes recurrent urinary tract infections with limited treatment options, has been shown to be susceptible to small molecular weight FimH inhibitor 4'-(a-d-mannopyranosyloxy)-*N*,3'-dimethylbiphenyl-3-carboxamide and results in reduced colonization of the bladder in murine models of urinary tract infections (UTI) even upon treatment of established infections [71]. While FimH antagonists may be limited to treatment of *E. coli* infection, anti-adhesion therapy targeting bacterial MAMs adhesion to host cells may lead to therapies with broader efficacy. Recombinant MAM7 from *V. parahaemolyticus* coupled to polymer beads has been shown to inhibit bacterial adhesion in a wide range of Gram-negative infections, including antibiotic resistant strains isolated from the wounds of wounded military personnel [72,73].

## 5. Summary

Recent advances have further highlighted the prospect of targeting bacterial adhesion as a viable method to treat a broad range of bacterial infections and with the rise of multidrug resistant bacteria presenting an ever increasing problem the need for the development of novel therapies is of the upmost importance. Although the molecular mechanisms of many bacterial adhesins are known, new adhesin classes have been found in recent years for which more work is still needed to define their molecular interactions. We note that especially interactions between adhesins and carbohydrate-based host cell ligands, while abundantly represented in nature, are still not well understood in terms of the effect these interactions have on host cellular signaling. With new advances in the application of chemical biology approaches to the study of bacterial adhesion, it has become increasingly clear that in many cases, the function of bacterial adhesins transcends beyond physical attachment and has a direct impact on early signaling events during host–pathogen interactions and thus may facilitate bacterial colonization and spread. This information needs to be further utilized to develop more efficient therapies that target bacterial adhesion while avoiding off-target effects on the host.
